# Folate deficiency modifies the risk of CIN3+ associated with DNA methylation levels: a nested case–control study from the ASCUS-COL trial

**DOI:** 10.1007/s00394-023-03289-4

**Published:** 2023-12-21

**Authors:** María C. Agudelo, Samuel Agudelo, Attila Lorincz, Arianis Tatiana Ramírez, Kelly Melisa Castañeda, Isabel Garcés-Palacio, Arnold H. Zea, Chandrika Piyathilake, Gloria Ines Sanchez

**Affiliations:** 1 Infection and Cancer Group, School of Medicine, Universidad de Antioquia, Carrera 51D No 62-29, 050010 Medellín, Colombia; 2 Centre for Cancer Prevention, Wolfson Institute of Population Health, Queen Mary University of London, London, UK; 3 Epidemiology Group, School of Public Health, Universidad de Antioquia, Medellín, Colombia; 4 Stanley S. Scott Cancer Center, Microbiology, Immunology and Parasitology, LSU Health Sciences Center, New Orleans, USA; 5 Department of Nutrition Sciences, The University of Alabama at Birmingham, Birmingham, USA; 6 Present Address: Postdoctoral Fellow at the Prevention and Implementation Group, International Agency for Research On Cancer/World Health Organization, Lyon, France

**Keywords:** Folate, DNA methylation, HPV, Cervical cancer

## Abstract

**Purpose:**

To our knowledge, there are very few studies evaluating if the levels of folate modify the risk of cervical intraepithelial neoplasia grade 2 and higher (CIN2+ and CIN3+) associated with the levels of HPV genome methylation, two cofactors related to single carbon metabolism and independently associated with cervical cancer in previous studies. We conducted a case–control study nested in a three-arm randomized clinical pragmatic trial (ASCUS-COL trial) to evaluate the risk of CIN3+ associated with methylation levels according to serum folate concentrations.

**Methods:**

Cases (*n* = 155) were women with histologically confirmed CIN2+ (113 CIN2, 38 CIN3, and 4 SCC) and controls were age and follow-up time at diagnosis-matched women with histologically confirmed ≤ CIN1 (*n* = 155), selected from the 1122 hrHPV + women of this trial. The concentrations of serum folate were determined by the radioimmunoassay SimulTRAC-SNB-VitaminB12/Folate-RIAKit and the methylation levels by the S5 classifier. Stepwise logistic regression models were used to estimate the association between folate or methylation levels and CIN2+ or CIN3+. The joint effect of folate levels and methylation on the risk of CIN3+ was estimated using combinations of categorical stratifications.

**Results:**

Folate levels were significantly lower in women with CIN3+ than in other diagnostic groups (*p* = 0.019). The risk of CIN3+ was eight times higher (OR 8.9, 95% CI 3.4–24.9) in women with folate deficiency and high methylation levels than in women with normal folate and high methylation levels (OR 1.4, 95% CI 0.4–4.6).

**Conclusion:**

High methylation and deficient folate independently increased the risk of CIN3+ while deficient folate combined with high methylation was associated with a substantially elevated risk of CIN3+.

**Supplementary Information:**

The online version contains supplementary material available at 10.1007/s00394-023-03289-4.

## Introduction

A persistent infection with oncogenic or high-risk genotypes of human papilloma virus (hrHPV) is necessary for the development of high-grade precancerous lesions, such as cervical intraepithelial neoplasia (CIN) grade 3 and cervical cancer (CC) [1–3]. Abnormal DNA methylation appears to be a key determinant of HPV infections that proceed to CIN3 and invasive cancer (CIN3+). Several studies have shown that cervical cancer can be predicted years in advance by increased levels of methylation in the HPV genome. A key metabolite associated with normal cellular methylation pathways is folate. Lower folate levels have been associated with the risk of developing cancers in several organs that include CC [4]. Thus, we were interested to see if serum levels of folate may correlate with levels of DNA methylation to predict even higher risk for CIN3 and invasive cervical cancer than hyper-methylation alone.

Using a cohort of women characterized for 37 genotypes of HPVs, biopsy-based CIN diagnoses, and other risk factors, a research team from the University of Alabama at Birmingham documented for the first time that women with lower circulating concentrations of folate who were positive for any HR-HPV genotype were twice as likely to have primary CIN2+ lesions, while HPV-16 positive women with lower folate levels were nine times more likely to have CIN2+ [5].Thereafter several but not all studies have confirmed this association [6–12]. Folate is a micronutrient essential in the synthesis of S-adenosylmethionine (SAM) [13, 14], which is the main donor of methyl groups for DNA methylation [15]. DNA methylation is an epigenetic modification which plays a key role in genome organization, suppression of viruses and invasive/mobile DNA, and in the regulation of transcription. Aberrant methylation patterns are observed in a large and diverse number of human cancers [16]. Consequently, malfunction of several factors or alteration of methyl-donor molecules’ (folic acid and S-adenosylmethionine) availability can contribute simultaneously to DNA methylation changes and cancer [17].

DNA methylation depends on the transfer of methyl groups to DNA-by-DNA methyltransferases DNMT1, DNMT3a, and DNMT3b [18, 19]. The role of HPV-induced epigenetic changes in cervical carcinogenesis is supported by several studies. Transfection of human foreskin keratinocytes (HFK) with HPV16 and HPV18 genomes induces overexpression of DNMT1 and DNMT3B [20]. On the other hand, there is in vitro evidence that differential methylation levels of CpG dinucleotides within the binding sites of the viral E2 protein, which in turn regulates the expression of E6/E7 oncoproteins, determine HPV-mediated cell transformation [21]. The hrHPV-induced epigenetic modifications including hyper-methylation of the HPV L1 and L2 and of host genes are associated with a higher probability of progression to CIN3+ in hrHPV-positive women [22–24]. In a study conducted among women with ASCUS cytology, we observed that the levels of S5 classifier, a test that detects methylation of regions of HPV L1 and L2 genes of HPV16, 18, 31, and 33 in combination with the methylation of the promoter of the human *EPB41L3* gene, increased significantly with the severity of the histopathological diagnosis (Cuzick trend test *χ*^2^ = 42.6, *p* < 0.001) [25]. However, to our knowledge, there are very few studies evaluating if the levels of folate modify the risk of CIN3+ associated with the levels of HPV and host genome methylation [26]. In this study, we evaluated the risk of CIN3+ as well as CIN2 (an occasional indicator of concomitant or subsequent CIN3), collectively known as CIN2+, associated with L1/L2 and *EPB41L3* methylation levels according to serum folate concentrations in the previously studied group of women.

## Methods

### Study design

We conducted a case–control study nested in a three-arm randomized clinical pragmatic trial, (The ASCUS-COL trial). Cases were all women with a histopathologically confirmed CIN2+ or CIN3+ diagnosis at any time during the ASC-US-COL trial. Controls were women with a histopathologically negative or CIN1 diagnosis, matched 1:1 by age (± 5 years) and time to diagnosis (± 6 months) to each case. Because the strong evidence about hrHPV infection as a necessary step in the development of CIN2+ and CIN3+, the selection of participants was restricted to 1122 hrHPV-positive women at recruitment. Briefly, the ASCUS-COL is a pragmatic clinical trial that compared under routine conditions of opportunistic CC screening, the effectiveness of immediate colposcopy, conventional cytology at 6 and 12 months, and the HPV test to detect CIN2+ after 24 months of follow-up. This trial included 2661, 20–69-year-old women with first time ASC-US cytology (atypical squamous cells of undetermined significance) recruited between January 2011 and January 2014 from the screening services of three Healthcare Management Organizations (HMOs) that provided their services in the city of Medellín, Colombia. Follow-up was carried out for 2 years from the initial date of recruitment and at each visit, the clinical staff filled out a questionnaire that included sociodemographic data and CC risk factors. A pelvic examination was conducted, and exfoliated cervical cells were collected for conventional cytology and hrHPV testing as well as peripheral blood sampling for metabolite determination. To detect the CIN2+ lesions undiagnosed during the previous 24 months, all women, regardless of the clinical management strategy assigned at the recruitment visit, underwent a 24-month follow-up exit visit for hrHPV and conventional cytology testing. Women negative for both tests had a safe exit from the study while those who had HPV and/or ≥ ASC-US cytology were referred for a strictly controlled colposcopy-directed/biopsy procedure performed by trained clinical staff, and the quality of this procedure was independently ensured by the clinical director in-charge of the study. During colposcopy, two biopsies of the observed lesions and a random biopsy of the apparently healthy cervical epithelium were taken. If no obvious lesions were observed, two random biopsies were taken from the apparently “normal” epithelium. Histopathological diagnoses were assigned by a certified pathology laboratory and confirmed by a panel of two expert pathologists external to these laboratories. In the ASCUS-COL study, women were excluded if they had abnormal cytology within the last year (as it could suggest that the index cytology was a follow-up rather than a screening test), large loop excision of the transformation zone (LLETZ), and/or hysterectomy. Additionally, women were excluded if they were not mentally able to provide informed consent, were pregnant, HIV-positive or with other immunosuppressive conditions, or planning to move out of the study area [27].

### Selection of cases and controls

**Fig. 1:**
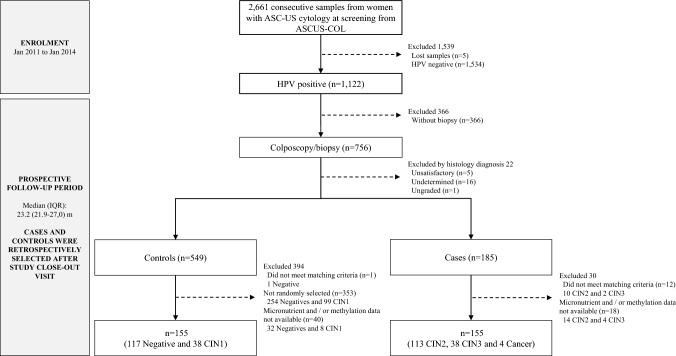
Flowchart. This diagram indicates the number of women with ASC-US cytology included in the ASCUS-COL trial. We selected all CIN2+ cases diagnosed at any time during the clinical trial. Controls were selected among women of the trial that remained free of disease (negative and/or CIN1verified by colposcopy AND biopsy) during the study and 1:1 paired to cases by age and time to colposcopy/biopsy. Women were HPV tested at recruitment in the HPV arm only and samples collected from women of the other 2 arms were all blindly tested after close-out study visit. All cases and controls were selected among HPV positive women. All cases and controls have similar chance for follow-up and received diagnosis and treatment if required

Figure 1 shows the flowchart for selection of cases and controls from women of the ASCUS-COL trial [27]. There were 185 cases (137 CIN2, 44 CIN3, and 4 SCC) and 549 controls (404 biopsies negative and 145 CIN1) diagnosed among the 1122 hrHPV-positive women with complete disease ascertainment after 24-month follow-up. Thirty cases (24 CIN2 and 6 CIN3) did not have folate and/or S5 methylation assay results and therefore these cases and their respective controls were excluded from the study. Finally, folate concentrations and methylation levels were determined in 155 CIN2+ cases (113 CIN2, 38 CIN3, and 4 CC cases) and 155 < CIN2 controls (117 negative and 38 CIN1). All procedures for data collection and analysis, treatment/assistance, or testing were conducted blinded at the end of the ASCUS-COL trial. Quality control of follow-up, disease ascertainment, histopathological diagnosis, for hrHPV, and S5 methylation testing have been described in previous publications of the ASCUS-COL trial [25, 27].

### Sample size

Sample size was estimated with R (version 4.0.3) using the epi.sscc function from the epiR version 2.0.19 package following methods for matched case–control studies as described [28]. A sample size of 175 cases and 175 controls was estimated for a two-sided 5% significance level and 80% power assuming a proportion of discordant pairs (*p*_dis_ = *s* + *t*) of 0.40, and odds ratio for discordant pairs (OR = *s*/*t*) of 2.0. We observed a proportion of discordant pairs (*p*_dis_ = *s* + *t*) of 0.37 in the post hoc analysis.

### Sample collection and identification of HPV infection

Cervical samples collected from the women using a cytobrush (HC Cervical Sampler) were subsequently transferred to a tube containing 1 ml of Specimen Transport Medium™ (STM). The Hybrid Capture 2 (HC2) assay from Qiagen HPV (QIAGEN, Gaithersburg, MD, USA) was used to detect HPV DNA. This DNA–RNA hybridization assay qualitatively detects a set of 13 hrHPV genotypes (16, 18, 31, 33, 35, 39, 45, 51, 52, 56, 58, 59, and 68), and was performed according to the manufacturer’s instructions at the facilities of the Infection and Cancer Group at the University of Antioquia. Relative Light Units (RLU) greater than or equal to 1 (≥ 1) were considered positive results. Quality controls are supplied with the HC2 HPV DNA Test. These controls were included in each assay, and the RLU/CO of each control must be within the acceptable ranges for it to be considered valid. Version 4.01 and higher Digene assay protocols for HPV automatically invalidate an assay if the controls are not within the specified limits. This test does not inform specific hrHPV genotypes.

### Extraction of DNA

DNA for the methylation assay was extracted using a 200 µL sample taken from the residual content of cervical cells stored at − 30 °C in the denatured STM tube to perform the HC2 test. Extraction details have been previously described [25, 29]. The extracted DNA was stored at − 30 °C and shipped frozen on dry ice to Queen Mary University of London where the classifier S5 methylation test was performed by a trained and validated scientist from the Medellin HPV team (ATR).

### DNA methylation test (classifier S5)

The methylation assays were based on end-point PCR and quantitative pyrosequencing of amplicons using primers for 6 target regions that cover a total of 22 CpG positions of the human *EPB41L3* gene and the late regions (L1 and L2) of HPV16, HPV18, HPV31, and HPV33. The S5 DNA methylation test has been characterized extensively in more than ten large studies and formally validated, details of the methylation test have been previously described [25, 30, 31].

### Folate measurement

The folate concentration was estimated in serum samples collected at the baseline of the ASCUS-COL study. Samples were taken at the time of recruitment of the participants by venipuncture in a dry 10 mL vacutainer tube. The blood samples were centrifuged at 2000 revolutions per minute (rpm) for 10 min within 20 min after sample collection, and the sera were immediately stored at − 80 °C. For quantification, the samples were sent in dry ice to the city of Birmingham, USA. Once there, the samples were thawed at room temperature. Total serum folate concentration was determined with the SimulTRAC-SNB Vitamin B12/Folate RIA Kit using previously established and validated protocols in the Laboratory of Nutritional Sciences at the University of Alabama at Birmingham [32].

### Multivariable analyses, outcomes, and confounding variables

According to the natural history of the disease and its relationship with our selected outcomes of CIN2+ and CIN3+, methylation levels were assumed as the exposure, the folate levels as the interaction variable and lifetime sexual partners, age at first intercourse, parity, and use of hormonal contraceptives were considered as potential confounders. There were few participants with past or current tobacco exposure, and therefore this variable was excluded. Folate concentrations were categorized as normal (≥ 6 to 20 ng/mL) and deficient (< 6 ng/mL) according to the recommendations of World Health Organization (WHO) [33]. Methylation levels were categorized as high (≥ 2.8) or normal (< 2.8) according to the cut-off point for the upper quartile of methylation levels among controls.

### Statistical analysis

Pearson’s Chi-square, Student’s *t*, and Mann–Whitney *U* tests were used to compare proportions, means, and medians between cases and controls, respectively. Conditional and unconditional stepwise logistic regression models were used to estimate the odds ratios (OR) with their corresponding 95% confidence intervals to test the association between folate or methylation levels and CIN2+ or CIN3+. These were fitted using all available variables or combination of selected variables to investigate different scenarios and then compared by the likelihood ratio (LR) test. The joint effect of folate levels and methylation was estimated using the following combinations of categorical stratifications: (1) reference category serum folate ≥ 6 to 20 ng/mL (normal) and methylation < 2.8 (normal), (2) serum folate < 6 ng/mL (deficient) and methylation < 2.8 (normal), (3) serum folate ≥ 6 to 20 ng/mL (normal) and methylation ≥ 2.8 (high), and (4) serum folate < 6 ng/mL (deficient) and methylation ≥ 2.8 (high). Models testing the joint effects were adjusted by same confounding variables stated previously. All statistical analyses were performed using the statistical software R version 4.0.3 (Development Core Team R: A language and environment for statistical computing. R Foundation for Statistical Computing, Vienna, Austria. ISBN 3-900051-07-0, URL http://www.r-project.org).

### Ethical considerations

Our study complied with Colombian Resolution 8430 of 1993 for studies in humans and followed CIOMS guidelines [34], the ethics committees for human experimentation of Sede de Investigación Universitaria (SIU) (Resolution 08-036-171) and School of Medicine Resolution 004/2008). These review boards approved this study (Resolution 12-40-473) and, therefore, it has been carried out in accordance with the ethical standards established in the Declaration of Helsinki of 1964 and its subsequent amendments. All participants signed informed consent and authorization to use samples and data for future research.

## Results

### Participant characteristics

Table 1 describes the sociodemographic characteristics and risk factors for women with diagnoses ≤ CIN1, CIN2+, and CIN3+. We observed statistically significant differences in variables between ≤ CIN1 and CIN2+ that were essentially in keeping with previously published results [35]. For example, age at first intercourse was a modest risk factor compared to the controls (*p* = 0.043). Similarly, parameters were for lifetime sexual partners (*p* = 0.045), parity (*p* = 0.039), use of hormonal contraceptives (*p* = 0.020). Median of methylation was also statistically different between ≤ CIN1 and CIN2+ or ≤ CIN1 and CIN3+ (*p* = 0.00001). Meanwhile, the median serum folate concentration was lower in CIN3+ compared to ≤ CIN1 (*p* = 0.039).

**Table 1:** Distribution of sociodemographic characteristics and risk factors for ≤ CIN1, CIN2+, and CIN3+

Characteristics	Total	≤ CIN1	CIN2+	*p* value	CIN3+	*p* value
	*N* = 310	*N* = 155	*N* = 155		*N* = 42	
	*n* (%)	*n* (%)	*n* (%)		*n* (%)	
Age (years)						
Mean [SD]	32.2 [9.5]	32.1 [9.5]	32.2 [9.6]	0.848	32.2 [9.6]	0.956
20–30	162 (52.3)	81 (52.3)	81 (52.3)		21 (50.0)	
31–40	88 (28.4)	41 (26.4)	47 (30.3)		12 (28.6)	
≥ 41	60 (19.3)	33 (21.3)	27 (17.4)		9 (21.4)	
Marital status						
Married/common law	128 (41.3)	55 (35.5)	73 (47.1)	0.106	22 (52.4)	0.134
Divorced/separated/widowed	22 (7.1)	13 (8.4)	9 (5.8)		3 (7.1)	
Single	160 (51.6)	87 (56.1)	73 (47.1)		17 (40.5)	
Education level						
Higher education	94 (30.3)	49 (31.6)	45 (29.1)	0.855	19 (45.2)	0.256
Secondary	112 (36.1)	54 (34.8)	58 (37.4)		12 (28.6)	
None or primary	104 (33.6)	52 (33.6)	52 (33.6)		11 (26.2)	
Age at first intercourse (years)						
Mean [SD]	17.3 [3.4]	17.7 [4.0]	16.8 [2.5]			
≥ 19	71 (22.9)	43 (27.7)	28 (18.1)	**0.043**	7 (16.6)	0.207
17–18	99 (31.9)	52 (33.6)	47 (30.3)		13 (31.0)	
≤ 16	140 (45.2)	60 (38.7)	80 (51.6)		22 (52.4)	
Lifetime sexual partners						
Median [IQR]	4.0 [4]	3.0 [4]	4.0 [3]			
1–2	90 (29.0)	53 (34.2)	37 (23.9)	**0.045**	9 (21.4)	0.114
≥ 3	220 (71.0)	102 (65.8)	118 (76.1)		33 (78.6)	
Parity						
Median [IQR]	1.0 [2]	1.0 [2]	1.0 [1]			
0	82 (26.5)	49 (31.6)	33 (21.3)	**0.039**	9 (21.4)	0.198
≥ 1	228 (73.5)	106 (68.4)	122 (78.7)		33 (78.6)	
Use of hormonal contraceptives						
Median [IQR]	3.0 [5.5]	3.0 [5.5]	3.5 [6.2]			
No	42 (13.6)	28 (18.1)	14 (9.0)	**0.02**	3 (7.1)	0.084
Yes	268 (86.4)	127 (81.9)	141 (91.0)		39 (92.9)	
Methylation levels (by S5)						
Median [IQR]	1.89 [6.1]	1.21 [2.2]	5.15 [9.0]	**0.00001**	9.21 [10.9]	**0.00001**
Folate levels						
Median [IQR]	5.80 [4.4]	6.00 [4.6]	5.63 [4.2]	0.505	4.66 [4.5]	**0.039**

Pearson’s Chi-square was used to compare the proportions of categorical variables and Student’s *t* and Mann–Whitney *U* tests, means, and medians of continuous variables (folates and methylation levels) between CIN2+ cases (113 with CIN2, 38 with CIN3 and 4 cancers), and ≤ CIN1 controls (117 women with negative biopsies and 38 with CIN1). The cut-off point of the methylation levels (classifier S5) was established according to the upper quartile of the methylation levels of the controls (≥ 2.8)

Significant values (*p* < 0.05) are shown in bold

### Serum folate levels

**Fig. 2:**
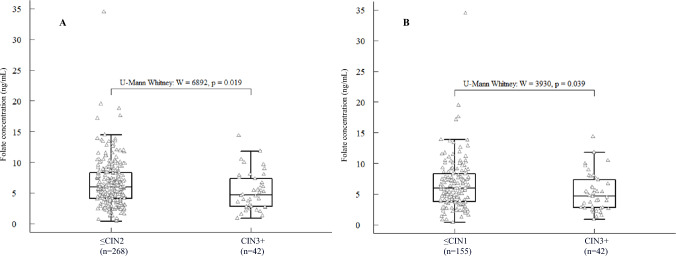
Comparison of folate distribution. **A** Comparison among women ≤CIN2 and CIN3. **B** Comparison among women ≤ CIN1 and CIN3+

The comparisons of folate levels between women without cervical lesions, those diagnosed with CIN1, CIN2, and CIN3+, are presented in Fig. 2. The median folate concentrations were 6.1 ng/mL, 5.8 ng/mL, 6.0 ng/mL, and 4.7 ng/mL for each of the groups. Folate levels were significantly lower in women with CIN3+ than in other groups (*p* = 0.019).

### Association between folate levels, methylation levels, and other risk factors with CIN2+ and CIN3+

Deficient folate (OR 2.03, 95% CI 1.01–4.24) and high methylation (OR 5.95 (95% CI 2.89–12.7) levels were associated with risk of CIN3+ in the univariate analysis, Supplementary Table 1. To assess the association between folate levels, methylation levels, and other risk factors, four distinct sets of variables were considered: (A) Methylation and use hormonal contraceptives; (B) Methylation, use of hormonal contraceptives, and marital status; (C) Methylation, use of hormonal contraceptives, marital status, and lifetime sexual partners; (D) Model C and folate level. Model D was the best multivariable model with LR *χ*^2^ (*df*) = 36.6 (6), Supplementary Table 1. We also constructed a stepwise model for all the variables using CIN2+ as the endpoint and found that only methylation was significant (data not shown).

### Association of high levels of L1, L2, and* EPB41L3* methylation with risk of CIN3+ according to serum folate concentrations

Compared with women who had normal folate and normal methylation levels, the risk of CIN3+ in women with deficient folate and normal methylation levels was not significantly different (OR 0.5, 95% CI 0.1–1.6); risk increased (but not significantly) in women with normal folate and high methylation (1.4, 95% CI 0.4–4.6). Meanwhile, the risk was significantly higher (OR 8.9, 95% CI 3.4–24.9) in women with deficient folate and high methylation levels. Therefore, the risk of CIN3+ in women who had deficient folate levels and high methylation was eight times that of women with normal folate levels and high methylation (Table 2). A greater joint effect predicted by the independent factors suggests that folate deficiency and high methylation contribute synergistically to the risk of CIN3+.

**Table 2:** Association of high levels of L1, L2 and EPB41L3 methylation with risk of CIN3+ according to serum folate concentrations

Folate level/methylation	≤ CIN1	CIN3+	Bivariate OR (IC95%)	*p* value	Multivariate OR (IC95%)	*p* value
	*N* = 155	*N* = 42				
Normal/normal	56	9	Ref		Ref	
Deficient/normal	60	5	0.5 (0.2–1.6)	0.571	0.5 (0.1–1.6)	0.237
Normal/high	22	5	1.4 (0.4–4.6)	0.264	1.4 (0.4–4.6)	0.628
Deficient/high	17	23	8.4 (3.4–22.6)	**9 × 10**^**−6**^	8.9 (3.4–24.9)	**1 × 10**^**−5**^

Odds ratio and 95% confidence intervals for association between CIN3+ and methylation levels according to serum folate concentrations. Unconditional stepwise logistic regression adjusted by marital status, lifetime sexual partners, and use of hormonal contraceptives. *p* for interaction 0.481

Significant values (*p* < 0.05) are shown in bold

## Discussion

We evaluated the risk of CIN3+ associated with methylation levels according to serum folate concentrations using a case–control study nested in a three-arm randomized clinical pragmatic trial, (ASCUS-COL trial) that included women from Medellin, Colombia. Serum folate levels were determined by standard radioimmunoassay [32] and methylation was assessed by the S5 classifier that accurately detects high-grade lesions of the cervix in hrHPV + women [25, 36].

In this analysis, we found that, compared to controls, a higher proportion of CIN2+ or CIN3+ cases have high methylation levels (S5 score ≥ 2.8), suggesting an independent effect of methylation on the severity of CIN lesions. Increased methylation in precancerous lesions of the cervix has been described in several studies for a decade, and they have had similar results even when different molecular biology techniques, epidemiological designs, and different regions of the viral and/or human genome have been used [22, 24, 25, 37].

The proportion of individuals with deficient folate levels found in our study (Table 1) is higher compared to data reported for other Latin American countries [38, 39] and similar to the one reported in 2015 in women of Medellín, Colombia. Considering that Colombia has had a national folate fortification plan for wheat flour since 1996, we expected to find normal or high levels of this micronutrient. However, we observed that folate levels were significantly lower when CIN3+ cases (*n* = 42) were compared with controls (Table 1 and Fig. 2, *U* Mann–Whitney *p* = 0.019). Although this same trend was observed in CIN2+, statistical significance was not reached, as expected, given our small numbers and because only a small proportion of CIN2+ represent true pre-cancers [40, 41]. This association between folate deficiency and the risk of precancerous lesions of the cervix and other cancer models has been documented for more than twenty years, especially in populations with limited economic resources [6, 7, 12, 42, 43].

We found that folate deficiency and high HPV DNA methylation are independentlyassociated with CIN3+ risk. As shown in Supplementary Table 1, both variables increased that risk when included in the multivariable model with the highest AIC value (LR *χ*^2^ (*df*) = 36.6 (6). Importantly, in the interaction analysis, we found that the risk of CIN3+ in women who had deficient folate levels and high methylation was eight times that of women with normal folate levels and high methylation (Table 2). Taken together, these observations, presented in Table Supplementary 1 and Table 2, favor a joint effect of folate deficiency and high methylation levels contributing synergistically to the risk of CIN3+. Few researchers have explored this interaction; however, Piyathilake et al. described findings in the same direction using a different epidemiological design and studying other regions of the viral genome [26].

We recognize several limitations of our study. Previous folate studies included hrHPV genotyping; however, the ASCUS-COL trial was a pragmatic trial that did not include genotyping due to Colombian recommendations for the management of women with ASC-US cytology at the time of this study. There was no adjustment for HPV status because cases and controls were chosen based on the positivity of the HC2 HPV test. We acknowledge that there exists a risk of selection bias because cases and controls may not be infected with the same viral genotype, and this influences the natural history of the disease and the risk of developing precancerous lesions. We measured folate levels in serum, which is considered an indicator of recent folate intake instead of red blood folate concentration that reflects more accurately the folate body status. The single measurement has the limitation that it is not possible to differentiate between a transitory decrease in dietary folate intake and chronic deficiency states. Although there is evidence that the general American population does not drastically change daily intake [44], similar data do not exist for the Colombian population. We also did not obtain information on the consumption of supplements, medications, or alcohol in these women, and products that affect folate metabolism. However, one of the exclusion criteria for the ASCUS-COL study was having chronic diseases such as autoimmune diseases or cancer. These criteria reduced the possibility of including participants who consumed anticonvulsants, barbiturates, methotrexate, or pyrimethamine, drugs that commonly decrease folate concentrations. Finally, the power achieved for the interaction analysis considering that the proportion of controls with deficient folate levels was 50%, and high methylation was 43%, with an estimated OR of 1.9, and a sample size of 155 cases and 155 controls was only 23.5%. For CIN3 analyses, the power achieved was 48.4%.

In conclusion, the results of this analysis indicate that even though Colombia has had a policy of fortifying wheat flour with folic acid for more than 20 years, the prevalence of folate deficiency among the population studied is higher than that reported in other Latin American countries and similar between cases and controls. We observed an association between methylation and the risk of CIN3+ in women with folate deficiency. Our analysis provides the first evidence from a Latin American country that folate deficiency may play an important role in modifying the associated risk between DNA methylation levels and CIN3+ risk. Methylation is the strongest predictor of CIN3+, especially in women who have deficient folate. High methylation appeared weakly but not significantly predictive for CIN3+ in women with normal folate, possibly due to small sample size. For future studies, we suggest additional and larger studies to confirm and refine our observations.

### Supplementary Information

Below is the link to the electronic supplementary material.

Supplementary file1 (DOCX 67 kb)

## Data Availability

The dataset, excluding personal identifiers, will be available to proper academic parties on request from the corresponding author in accordance with the data sharing policies of the Universidad de Antioquia.
