# Supplementary material for: Folate deficiency modifies the risk of CIN3+ associated with DNA methylation levels: a nested case–control study from the ASCUS-COL trial
Source: Eur J Nutr. 2023 Dec 21;63(2):563–72. doi: 10.1007/s00394-023-03289-4 (PMC10899296; doi:10.1007/s00394-023-03289-4)
Supplement: Supplementary file 1 — Supplementary file1 (DOCX 67 kb) [file 394_2023_3289_MOESM1_ESM.docx]

**Supplementary material**

**Supplementary Table 1. Association between folate levels and other risk factors with high-grade cervical lesions (CIN3+)**

| **Risk factor** | **≤CIN1** |  | **CIN3+** |  | **Univariate** | **p-value** | **Model A: Methylation + Use of hormonal**  **contraceptives** | **χ2** | **p-value** | **Model B: Model A + Marital status** | **χ2** | **p-value** | **Model C: Model B +** **Lifetime sexual partners** | | **χ2** | **p-value** | **Model D: Final model (Model C + Folate level** | **χ2** | **p-value** |
| --- | --- | --- | --- | --- | --- | --- | --- | --- | --- | --- | --- | --- | --- | --- | --- | --- | --- | --- | --- |
|  | **N = 155** |  | **N = 42** |  | **OR (95% CI)** |  | **OR (95% CI)** |  |  | **OR (95% CI)** |  |  | **OR (95% CI)** | |  |  | **OR (95% CI)** |  |  |
| **Methylation level (clasificador S5)** |  |  |  |  |  |  |  |  |  |  |  |  |  | |  |  |  |  |  |
| Normal (cut-off point <2.8) | 116 |  | 14 |  | 1.00 |  | 1.00 | 24.3 |  | 1.00 | 24.3 |  | 1.00 | | 24.3 |  | 1.00 | 24.3 |  |
| High (cut-off point ≥2.8) | 39 |  | 28 |  | **5.95 (2.89-12.7)** | **2*10^−6^** | **6.24 (2.25-13.2)** |  | **2*10^−6^** | **6.15 (2.89-13.1)** |  | **2*10^−6^** | **6.21 (2.89-13.3)** | |  | **2*10^−6^** | **5.99 (2.78-12.9)** |  | **5*10^−6^** |
| **Folate level** |  |  |  |  |  |  |  |  |  |  |  |  |  | |  |  |  |  |  |
| Normal (6-20 ng/mL) | 78 |  | 14 |  | 1.00 |  |  |  |  |  |  |  |  | |  |  | 1.00 | 2.0 |  |
| Deficient (<6 ng/mL) | 77 |  | 28 |  | **2.03 (1.01-4.24)** | 0.053 |  |  |  |  |  |  |  | |  |  | 1.81 (0.82-3.99) |  | 0.141 |
| **Age (years)** |  |  |  |  |  |  |  |  |  |  |  |  |  | |  |  |  |  |  |
| 20 – 30 | 81 |  | 21 |  | 1.00 |  |  |  |  |  |  |  |  | |  |  |  |  |  |
| 31 – 40 | 41 |  | 12 |  | 1.13 (0.49-2.49) | 0.767 |  |  |  |  |  |  |  | |  |  |  |  |  |
| ≥ 41 | 33 |  | 9 |  | 1.05 (0.42-2.48) | 0.910 |  |  |  |  |  |  |  | |  |  |  |  |  |
| **Marital status** |  |  |  |  |  |  |  |  |  |  |  |  |  | |  |  |  |  |  |
| Married/ common law | 55 |  | 22 |  | 1.00 |  |  |  |  | 1.00 | 3.1 |  | 1.00 | | 3.1 |  | 1.00 | 4.0 |  |
| Divorced/separated /widowed | 13 |  | 3 |  | 0.58 (0.12-2.01) | 0.424 |  |  |  | 0.54 (0.13-2.32) |  | 0.406 | 0.50 (0.12-2.16) | |  |  | 0.53 (0.12 – 2.28) |  | 0.393 |
| Single | 87 |  | 17 |  | 0.49 (0.24-1.00) | 0.050 |  |  |  | 0.51 (0.23-1.11) |  | 0.089 | 0.46 (0.21-1.03) | |  |  | 0.45 (0.20 – 1.00) |  | **0.049** |
| **Education level** |  |  |  |  |  |  |  |  |  |  |  |  |  | |  |  |  |  |  |
| Higher education | 52 |  | 19 |  | 1.00 |  |  |  |  |  |  |  |  | |  |  |  |  |  |
| Secondary | 54 |  | 12 |  | 0.61 (0.26-1.36) | 0.233 |  |  |  |  |  |  |  | |  |  |  |  |  |
| None or primary | 49 |  | 11 |  | 0.61 (0.26-1.40) | 0.255 |  |  |  |  |  |  |  | |  |  |  |  |  |
| **Age at first intercourse (years)** |  |  |  |  |  |  |  |  |  |  |  |  |  | |  |  |  |  |  |
| ≥19 | 43 |  | 7 |  | 1.00 |  |  |  |  |  |  |  |  | |  |  |  |  |  |
| 17-18 | 52 |  | 13 |  | 1.54 (0.58-4.40) | 0.402 |  |  |  |  |  |  |  | |  |  |  |  |  |
| ≤16 | 60 |  | 22 |  | 2.25 (0.92-6.13) | 0.089 |  |  |  |  |  |  |  | |  |  |  |  |  |
| **Lifetime sexual partners** |  |  |  |  |  |  |  |  |  |  |  |  |  | |  |  |  |  |  |
| 1-2 | 53 |  | 9 |  | 1.00 |  |  |  |  |  |  |  | 1.00 | | 2.9 |  | 1.00 | 2.2 |  |
| ≥3 | 102 |  | 33 |  | 1.91 (0.88-4.50) | 0.118 |  |  |  |  |  |  | 2.11 (0.87-5.13) | |  | 0.099 | 2.16 (0.89 – 5.26) |  | 0.089 |
| **Parity** |  |  |  |  |  |  |  |  |  |  |  |  |  | |  |  |  |  |  |
| 0 | 49 |  | 9 |  | 1.00 |  |  |  |  |  |  |  |  | |  |  |  |  |  |
| ≥1 | 106 |  | 33 |  | 1.69 (0.78-4.01) | 0.202 |  |  |  |  |  |  |  | |  |  |  |  |  |
| **Use of hormonal** |  |  |  |  |  |  |  |  |  |  |  |  |  | |  |  |  |  |  |
| **contraceptives** |  |  |  |  |  |  |  |  |  |  |  |  |  |  |  |  |  |  |  |
| No | 28 |  | 3 |  | 1.00 |  | 1.00 | 4.16 |  | 1.00 | 4.16 |  | 1.00 | | 4.16 |  | 1.00 | 4.2 |  |
| Yes | 127 |  | 39 |  | 2.87 (0.95-12.4) | 0.097 | 3.37 (0.92-12.36) |  | 0.067 | 3.34 (0.91-12.3) |  | 0.070 | 3.17 (0.85-11.8) | |  | 0.086 | 2.81 (0.74-10.60) |  | 0.127 |
| LR χ2 (df) |  |  |  |  |  |  | 28.4 (2) | | | 31.5 (4) | | | | 34.4 (5) | | | **36.6 (6)** | | |
| Odds ratio and 95% confidence intervals for the association between CIN3+ and risk factors. Unconditional stepwise logistic regression.  (df) = degrees of freedom. | | | | | | | | | | | | | | | | | | | |
